# Study of Speech Recognition in Noise and Working Memory in Adults and Elderly with Normal Hearing

**DOI:** 10.1055/s-0044-1779432

**Published:** 2024-02-16

**Authors:** Daniela Aiko Akashi, Maria Cecília Martinelli

**Affiliations:** 1 Department of Speech, Language and Hearing Sciences, Universidade Federal de São Paulo (UNIFESP), São Paulo, SP, Brazil

**Keywords:** working memory, speech recognition, aging, auditory processing

## Abstract

**Introduction** In clinical practice, patients with the same degree and configuration of hearing loss, or even with normal audiometric thresholds, present substantially different performances in terms of speech perception. This probably happens because other factors, in addition to auditory sensitivity, interfere with speech perception. Thus, studies are needed to investigate the performance of listeners in unfavorable listening conditions to identify the processes that interfere in the speech perception of these subjects.

**Objective** To verify the influence of age, temporal processing, and working memory on speech recognition in noise.

**Methods** Thirty-eight adult and elderly individuals with normal hearing thresholds participated in the study. Participants were divided into two groups: The adult group (G1), composed of 10 individuals aged 21 to 33 years, and the elderly group (G2), with 28 participants aged 60 to 81 years. They underwent audiological assessment with the Portuguese Sentence List Test, Gaps-in-Noise test, Digit Span Memory test, Running Span Task, Corsi Block-Tapping test, and Visual Pattern test.

**Results** The Running Span Task score proved to be a statistically significant predictor of the listening-in-noise variable. This result showed that the difference in performance between groups G1 and G2 in relation to listening in noise is due not only to aging, but also to changes in working memory.

**Conclusion** The study showed that working memory is a predictor of listening performance in noise in individuals with normal hearing, and that this task can provide important information for investigation in individuals who have difficulty hearing in unfavorable environments.

## Introduction

Speech comprehension depends on the integrity of the auditory nervous system and may suffer from the influence of the stimulus presentation, the type of response requested, and the individual's language experience. 1 2

Thus, it is observed that the basic audiological assessment, which involves obtaining pure tone auditory thresholds and tests with isolated words, does not make it possible to infer the communicative capacity of each individual as monosyllabic words, which are reproduced at uniform levels of presentation and do not suffer the effects of co-articulation, are not representative of the everyday speech to which the patient is exposed in everyday communication situations. 3 4

Thus, the tests chosen by different lists of clinical conditions are of great importance because they allow an analysis of the comprehension of the spoken message in circumstances close to the communication conditions that you encounter in your daily life. 5

To understand the role of cognition in the intelligibility of oral language, one of the pillars of cognition must be considered, the concept of working memory.

Working memory is important for language processing because it is responsible for interpreting, retaining information extracted from speech sounds, inhibiting, or ignoring irrelevant information, as well as compensating for peripheral distortions of sounds that are observed in hearing loss. This compensation is achieved by allocating more attentional resources. 6

In clinical practice, it is observed that patients with the same degree and configuration of sensorineural hearing loss, 7 or even with auditory thresholds within the normal range, 8 present substantially different speech perception performances. In addition to auditory sensitivity, researchers have demonstrated that working memory capacity, temporal processing ability, and processing speed interfere with speech perception. 9 10

Therefore, studies are needed to investigate the performance of listeners in unfavorable listening conditions to identify which processes can interfere with speech perception.

Thus, the objective of the present study was to verify the influence of age, temporal processing, and working memory on speech recognition in noise using the Portuguese Sentence List Test.

The hypothesis that guided the present study is that elderly people with normal hearing have worse speech recognition in noise than adults, due to the cognitive and temporal processing decline characteristic of aging.

## Methods

This is a prospective study with ethics research approved by the institutional ethics committee (n°0839/2019).

The participation of the individuals in the study was conditioned to their acceptance and signature of an informed consent form, prepared according to the recommendations of the National Health Council in compliance with the normative resolution 466/12, of the CNS/MS.

The eligibility criteria for the composition of the sample were:

- Adults aged between 19 and 59 years who presented hearing thresholds ≤ 25 dB HL in frequencies from 250 Hz to 8 kHz;
- Elderly aged 60 years or older with audiometric thresholds ≤ 25 dB HL in frequencies from 250 Hz to 4,000 Hz.
- All individuals exhibited no evident cognitive and/or psychiatric impairment.

Based on the eligibility criteria, 38 participants were selected for convenience and distributed into two groups according to age:

Adult group (G1): Included 10 adults, 10 of whom were female, aged between 21 and 33 years (mean 23.8 ± 3.42), with an educational background ranging from 14 to 22 years (mean 16.6 ± 2.63)

Elderly group (G2): Consisted of 28 elderly people, 23 females and 5 males aged between 60 and 81 years (mean 67.1 ± 6.41), with an educational background spanning from 3 to 22 years (mean 12 ± 4.49).

### Procedures

The tests were applied individually, in a quiet room, not exceeding 1 hour of application, and were conducted in 2 sessions.

All eligible individuals who consented to take part in the study underwent the following procedures:

**Socioeconomic Classification Criteria (** Brazilian Association of Population Studies – **ABEP** , in the Portuguese acronym **)** 11 **:** The Socioeconomic Classification Criteria – (ABEP) (2019) was applied to estimate the economic stratification of the studied population, based on income and education indicators, the use of essential public services, and the amount of household comfort goods. This instrument is objective and easy to apply, in which each investigated item has a corresponding value, and the sum of these values classifies individuals into different classes: A, B1, B2, C1, C2, and D/E. This classification reflects values, attitudes, social norms, lifestyles, and consumption patterns that vary by socioeconomic status.

**Audiological evaluation:** This evaluation included visual inspection of the external acoustic meatus, pure tone audiometry, which investigated hearing thresholds for pure tones ranging from 250 to 8,000 Hz in each ear, and speech audiometry. Speech audiometry involved the assessment of Speech Reception Threshold (SRT) and Word Recognition Score (WRS).

**Portuguese Sentence List Test (PSLT)**^12^ : The Sentence Recognition Threshold in Noise (SRTN) was investigated in free field and performed in an acoustically-treated environment. Participants were positioned 1 m from the sound source, at a 0° azimuth angle. The SRTN of the participants was determined using the sequential or adaptive strategy, or ascending-descending method 13 . This method determines the level at which an individual can correctly identify ∼ 50% of the speech stimuli presented at a given signal/noise ratio.

**Gaps-in-noise test (GIN)**^14^ : The test was applied in a free field at an intensity of 50 dB SL. The test involved a series of white noise stimuli tracks, each lasting 6 seconds, with gaps inserted at different positions and of varying durations (2, 3, 4, 5, 6, 8, 10, 12, 15 and 20 ms). Participants were instructed to raise their hand when they noticed a silent interval.

**Digit Span Test**^15^**:** This test assesses a subject's ability to retain information in the phonological subsystem of working memory, specifically in direct order. Increasing sequences of digits were displayed on a computer screen at a rate of one per second. At the end of each sequence, the participant was required to repeat the digits in the same order they were presented. The test ends when there is an error in two sequences with the same number of digits. The same methodology is applied to evaluate reverse digit span, with participants asked to repeat the sequences in reverse order. In this case, the goal is to assess executive functioning. The score is determined by the number of digits in the longest sequence remembered correctly.

**Running Memory Span**^16^**:** Lists of digits were presented, one by one, on a computer screen. Number sequences ranged from 5 to 20 digits, making it difficult to remember all the digits. The interval between stimuli was 500 ms. The participant was instructed to repeat the last remembered digits in the correct sequence.

**Corsi Block Test (CBT)**^17^**:** Test performed on a wooden board on which nine blocks of equal dimensions are arranged irregularly. During the test, the examiner touches a certain sequence of blocks, and shortly afterward, the subject is required to point to the blocks in the exact order that the examiner touched them. The difficulty was progressively increased by expanding the number of blocks in each sequence. The test ends when there is an error in two sequences with the same number of blocks. The score is determined by the highest number of blocks in a sequence that the subject accurately repeats.

**Visual Patterns test (TPV)**^18^**:** Evaluates visual working memory retention. Participants view matrices of varying sizes, each with half of the squares filled, for 3 seconds. The complexity level of a pattern is given by the number of filled cells in the grid and ranges from 2 (in the 2 × 2 matrix) to 15 (in the 5 × 6 matrix). Three different matrices of each size are shown. The test ends when two out of three presented matrices are missed. The score is based on the last correctly retrieved matrix.

### Statistical Method

The statistical significance value adopted was equal to 5% ( *p* ≤ 0.05). The IBM SPSS Statistics for Windows, version 25.0 (IBM Corp., Armonk, NY, USA) was used.

To calculate the 95% confidence intervals, the corrected and accelerated bias method was used based on 2,000 bootstrap samples.

To interpret the sizes of the effects, it is suggested to use the classification proposed by Cohen. 19

## Results

### Comparison of Groups Regarding Education, ABEP, GIN, Corsi, Visual Patterns test, Running Digits, Reverse Digits, and Sentence Recognition

Table 1 presents an analysis of the data distribution to verify whether they comply with the normality assumption, to assist in the decision of choosing the test to compare these variables (parametric or non-parametric test). The Shapiro-Wilk test was used to verify compliance with the normality assumption.

**Table 1:** Analysis of the distribution of data regarding education, score, ABEP, gap detection threshold of GIN, Corsi, TPV, Running, Digits, Reverse Digits, and Sentence Recognition (Average and S/R)

PAC	Group	Test statistics	Shapiro-Wilk *p* -value
Education	G1	0.843	0.048*
	G2	0.982	0.893
ABEP	G1	0.912	0.295
	G2	0.948	0.177
GIN	G1	0.834	0.037*
	G2	0.927	0.052
Corsi	G1	0.895	0.191
	G2	0.898	0.010*
TPV	G1	0.916	0.322
	G2	0.816	< 0.001*
Running	G1	0.971	0.899
	G2	0.951	0.216
Digits	G1	0.841	0.045*
	G2	0.930	0.063
Reverse Digits	G1	0.820	0.026*
	G2	0.882	0.005*
Sentence Recognition - Average	G1	0.848	0.056
	G2	0.819	< 0.001*
Sentence Recognition – S/R	G1	0.867	0.091
	G2	0.901	0.012*

*: Statistically significant value at the 5% level ( *p* ≤ 0.05).

For analyses involving at least one distribution that violated the normality assumption ( *p* -value ≤ 0.05), non-parametric tests were used.

Table 2 presents the central tendency and dispersion measures for schooling, ABEP, GIN, Corsi, TPV, Running, Digits, Reverse Digits, and Sentence Recognition across the groups. Group comparisons were conducted using either Student *t* -tests (parametric) or Mann-Whitney U-tests (non-parametric). Effect sizes were assessed using coefficients d 19 or r 20 .

**Table 2:** Descriptive values and comparative analysis of the groups in relation to education, ABEP, GIN, Corsi, TPV, Running, Digits, Reverse Digits, and Sentence Recognition

PAC	Group	n	Average	SD	Median	Min.	Max.	*p*	T.E.
Education	G1	10	16.60 [15.20. 18.20]	2.63	17.00 [14.00. 18.00]	14.00	22.00	0.003*	0.472 ^r^
	G2	28	12.00 [10.32. 13.71]	4.49	12.00 [11.00. 12.00]	3.00	22.00		
ABEP	G1	10	35.70 [29.80. 42.60]	9.31	34.50 [28.50. 41.00]	26.00	53.00	0.094 ^a^	0.639 ^d^
	G2	28	29.75 [26.39. 32.96]	9.41	29.00 [28.50. 29.50]	15.00	48.00		
GIN	G1	10	6.60 [5.70. 7.60]	1.78	6.00 [5.00. 8.00]	5.00	10.00	< 0.001*	0.567 ^r^
	G2	28	12.82 [11.04. 14.75]	5.59	12.00 [11.00. 12.00]	4.00	25.00		
Corsi	G1	10	6.20 [5.80. 6.60]	1.03	6.00 [5.50. 7.00]	5.00	8.00	0.011*	0.402 ^r^
	G2	28	5.07 [4.68. 5.46]	1.15	5.00 [5.00. 5.00]	3.00	8.00		
TPV	G1	10	7.70 [6.80. 8.60]	1.57	8.00 [8.00. 8.00]	5.00	10.00	< 0.001*	0.687 ^r^
	G2	28	4.61 [4.25. 5.00]	1.17	4.00 [4.00. 5.00]	3.00	8.00		
Running	G1	10	2.56 [2.17. 2.99]	0.73	2.55 [2.13. 3.05]	1.40	3.60	< 0.001* ^a^	1.450 ^d^
	G2	28	1.50 [1.21. 1.77]	0.69	1.65 [1.18. 1.85]	0.30	2.90		
Digits	G1	10	7.80 [6.90. 8.60]	1.23	8.00 [8.00. 8.00]	5.00	9.00	0.001*	0.530 ^r^
	G2	28	5.68 [5.07. 6.32]	1.54	5.50 [5.00. 6.00]	3.00	9.00		
Reverse Digits	G1	10	6.90 [6.40. 7.40]	0.88	7.00 [7.00. 7.00]	5.00	8.00	< 0.001*	0.701 ^r^
	G2	28	4.54 [4.25. 4.86]	1.00	5.00 [5.00. 5.00]	3.00	6.00		
Sentence Recognition - Average	G1	10	46.16 [45.12. 47.10]	1.94	46.95 [44.60. 47.75]	43.20	48.05	< 0.001*	0.544 ^r^
	G2	28	51.90 [49.98. 54.03]	6.49	51.60 [50.30. 52.20]	40.60	72.50		
Sentence Recognition – S/R	G1	10	1.32 [0.30. 2.25]	1.82	1.95 [0.40. 2.75]	-1.80	3.05	< 0.001*	0.544 ^r^
	G2	28	5.48 [4.26. 6.64]	3.72	6.20 [5.10. 7.20]	-4.40	11.20		

Abbreviations: Max, maximum; Min, minimum; SD, standard deviation.

Student *t* -test for independent samples (a) and Mann-Whitney U test (b).

*: Statistically significant value at the 5% level ( *p* ≤ 0.05); S.E.: effect size; Note: For the gap detection threshold of the GIN test, thresholds above 20 milliseconds were coded as 25 milliseconds.

The results in Table 2 indicate statistically significant differences between the groups in terms of schooling, GIN, Corsi, TPV, Running Digits, Reverse Digits, Sentence Recognition – average, and Sentence Recognition - S/N. For the variables schooling, Corsi, TPV, Running, Digits, and Reverse Digits, G1 had a higher value compared with G2. For the variables GIN, Sentence Recognition – average, and Sentences Recognition - S/N, G2 presented higher values in comparison to the G1.

### Investigation of the Predictive Capacity of Schooling, ABEP, GIN, Corsi, TPV, Running, Digits, and Reverse Digits in Relation to Sentence Recognition

Tables 3 and 4 display multiple linear regression models developed to check the predictive capabilities of various factors of listening in noise (Sentence Recognition - average, and Sentence Recognition - S/N) for the entire study sample. The models in Tables 3 and 4 use Sentence Recognition - average and Sentence Recognition - S/R as dependent variables, respectively. The independent variables include the ability of age group (group), education, socioeconomic status (ABEP), temporal processing (GIN), and working memory (Corsi, TPV, Running, Digits, and Reverse Digits) in relation to listening in noise (Sentence Recognition – average, and Sentence Recognition - S/N) for the total study sample. The models in Tables 3 and 4 had Sentence Recognition - average and Sentence Recognition - S/R as dependent variables, respectively, and group, schooling, ABEP score, GIN gap detection threshold, and scores on the Corsi, TPV, Running, Digits, and Reverse Digits tests as independent variables.

**Table 3:** Multiple regression linear model of age, education, socioeconomic status, temporal processing and working memory as predictors of listening in noise (sentence recognition – average)

Step		b	β	*p*
1	Constant	40.41 [37.05. 43.66]	–	< 0.001*
	Group	5.75 [3.06. 8.59]	0.42	0.010*
2	Constant	33.93 [20.24. 43.86]	–	< 0.001*
	Group	7.15 [3.45. 12.02]	0.52	0.004*
	Education	0.31 [-0.08. 0.82]	0.22	0.191
3	Constant	36.01 [22.02. 46.43]	–	< 0.001*
	Group	7.04 [3.52. 11.31]	0.51	0.005*
	Education	0.42 [-0.13. 1.05]	0.31	0.113
	Score ABEP	-0.11 [-0.38. 0.11]	-0.17	0.341
4	Constant	35.19 [21.52. 47.29]	–	< 0.001*
	Group	6.58 [2.87. 10.44]	0.48	0.014*
	Education	0.46 [-0.26. 1.24]	0.34	0.101
	Score ABEP	-0.11 [-0.37. 0.09]	-0.17	0.345
	Gap detection threshold – GIN	0.10 [-0.49. 0.86]	0.09	0.620
5	Constant	36.61 [5.90. 62.05]	–	0.017*
	Group	5.21 [-2.40. 14.22]	0.38	0.219
	Education	0.56 [-0.36. 1.63]	0.41	0.058
	Score ABEP	-0.12 [-0.38. 0.10]	-0.18	0.368
	Gap detection threshold – GIN	-0.04 [-0.58. 0.67]	-0.04	0.857
	Corsi	1.35 [-0.34. 3.33]	0.27	0.111
	TPV	-0.99 [-3.36. 0.84]	-0.30	0.262
	Running	-3.52 [-7.56. 0.89]	-0.48	0.024*
	Digits	0.09 [-1.56. 1.50]	0.03	0.926
	Reverse Digits	1.01 [-1.88. 4.13]	0.23	0.443

Step 1: r ^2^ = 0.172, r ^2^ adjust. = 0.149 ( *p* = 0.010); Step 2: r ^2^ = 0.212, r ^2^ adjust. = 0.167 ( *p* = 0.015); Step 3: r ^2^ = 0.233, r ^2^ adjust. = 0.165 ( *p* = 0.027); Step 4: r ^2^ = 0.239, r ^2^ adjust. = 0.147 ( *p* = 0.055); Step 5: r ^2^ = 0.427, r ^2^ adjust. = 0.243 ( *p* = 0.043).

*: Statistically significant value at the 5% level ( *p* ≤ 0.05).

**Table 4:** Multiple regression linear model of age, education, socioeconomic status, temporal processing and working memory as predictors of listening in noise (sentence recognition - S/N)

Step		b	β	*p*
1	Constant	-2.84 [-5.62. -0.15]	–	0.206
	Group	4.16 [2.37. 5.89]	0.49	0.002*
2	Constant	-4.50 [-11.88. 2.43]	–	0.227
	Group	4.52 [2.49. 6.70]	0.53	0.003*
	Education	0.08 [-0.19. 0.36]	0.09	0.572
3	Constant	-5.18 [-13.65. 2.56]	–	0.195
	Group	4.56 [2.45. 6.82]	0.54	0.003*
	Education	0.04 [-0.26. 0.31]	0.05	0.802
	Score ABEP	0.04 [-0.09. 0.19]	0.09	0.605
4	Constant	-4.98 [-13.35. 4.57]	–	0.234
	Group	4.67 [2.12. 7.13]	0.55	0.005*
	Education	0.03 [-0.35. 0.36]	0.04	0.856
	Score ABEP	0.04 [-0.09. 0.16]	0.09	0.609
	Gap detection threshold – GIN	-0.03 [-0.44. 0.36]	-0.04	0.837
5	Constant	-5.48 [-26.97. 9.62]	–	0.542
	Group	3.99 [-1.89. 10.60]	0.47	0.128
	Education	0.06 [-0.27. 0.37]	0.07	0.747
	Score ABEP	0.00 [-0.17. 0.20]	0.00	0.989
	Gap detection threshold – GIN	-0.07 [-0.49. 0.35]	-0.11	0.592
	Corsi	0.83 [-0.32. 1.92]	0.27	0.112
	TPV	-0.44 [-1.66. 1.15]	-0.22	0.417
	Running	-2.16 [-4.55. 1.15]	-0.48	0.024*
	Digits	0.51 [-0.41. 1.51]	0.23	0.401
	Reverse Digits	0.32 [-1.54. 2.15]	0.12	0.693

Step 1: r ^2^ = 0.240, r ^2^ adjust. = 0.219 ( *p* = 0.002); Step 2: r ^2^ = 0.247, r ^2^ adjust. = 0.204 ( *p* = 0.007); Step 3: r ^2^ = 0.253, r ^2^ adjust. = 0.188 ( *p* = 0.018); Step 4: r ^2^ = 0.254, r ^2^ adjust. = 0.164 ( *p* = 0.041); Step 5: r ^2^ = 0.425, r ^2^ adjust. = 0.240 ( *p* = 0.045).

*: statistically significant value at the 5% level ( *p* ≤ 0.05).

The independent variables were entered hierarchically in five steps. This approach of inserting independent variables was adopted to separately examine the effects of age group, education, socioeconomic status, temporal processing, and working memory on listening in noise.

The results of Tables 3 and 4 reveal that the Group variable was able to explain respectively 14.9% (adjusted r ^2^ = 0.149) of the variance observed in the Sentence Recognition - average and 21.9% (adjusted r ^2^ = 0.219) of the variance observed in the Sentence Recognition – S/R. No improvements were observed in the predictive capacity of the model in steps 2, 3, and 4. Throughout these steps, the Group variable remained a statistically significant predictor for both Sentence Recognition - average and Sentence Recognition - S/R. These findings demonstrate that schooling, socioeconomic status, and temporal processing did not significantly predict listening in noise. Furthermore, they demonstrate that the influence of age on listening in noise cannot be explained by these three variables.

In the fifth step, the variable Group' ceased to be a statistically significant predictor of the variable Sentence Recognition - average and Sentence Recognition - S/R. Instead, the Running test score proved to be a statistically significant predictor of this variable, explaining 24.3% and 24.0% of the variance, respectively. These findings suggest that the influence of age group on listening in noise is mediated by working memory, since controlling for the variables related to working memory led to the disappearance of the influence of the variable Group on the variables Sentence Recognition - average and Sentence Recognition - S/R. The unstandardized coefficients (b) indicate the change in the dependent variable (measured in units of each variable) when one unit is added to the independent variable's value, while all other variables are held constant. The standardized coefficients (β) indicate the change in standard deviations observed in the dependent variable when one standard deviation is added to the independent variable's value, with all other variables kept constant.

## Discussion

In the comparative analysis between the groups presented in Table 2 , significant differences were observed in terms of education, ABEP, GIN, Corsi, TPV, Running, Digits, Reverse Digits, SRTN, and Signal/Noise ratio - S/R.

The adult group had a longer average formal education duration than the older adult group. This finding may have influenced test performance in the different groups, as higher educational levels tend to positively impact cognitive task performance. 21 Pliatsikas et al. 22 investigated the influence of gender and education on working memory in older adults aged 58 to 89 years, and that age negatively affects working memory, while education has a positive association. Their research shows that aging has a detrimental effect on working memory, while years of formal education may lead to better performance in tasks involving working memory. Additionally, the correlation between schooling and performance in tests that involve temporal processing has already been observed. 21

Furthermore, it is noteworthy that the participants in the older adult group belonged to a lower socioeconomic level, which may have contributed to the low level of education found in this group. This relationship between socioeconomic status and education is consistent with previous research. For instance, a study by Foss, Formigheri, and Speciall 23 found significant differences in the cognitive aging process, as assessed through neuropsychological evaluations, of healthy elderly individuals in Brazil. This research highlights the role of socioeconomic factors in intensifying cognitive differences among the elderly.

The results of the tests indicate that adults outperformed the elderly in all tasks, aligning with previous studies that demonstrated better speech recognition performance in noise among younger listeners with normal hearing compared with older listeners. 8 10 Such performance can be attributed to age-related declines in hearing function and concurrent cognitive changes that can impact speech understanding. During speech recognition in noise, the individual needs mainly memory and attention to be able to remember speech information, while filtering out irrelevant information. 7

In tasks assessing working memory, substantial performance difference was observed between the groups in the TPV, Running, Digits and –Reverse Digits tests. However, the Corsi Block test was the only one which showed an average difference. These findings support the notion that advancing age is linked with changes in cognitive abilities, involving a decline in working memory performance. 10

In the present study, we found a large difference in performance between the groups in the GIN test, with an effect size of 0.567r. Specifically, G1 exhibited a significantly lower average detection threshold for silence intervals (6.60 ms) when compared with group G2 (12.82 ms). Therefore, these findings indicate that younger adults performed better in temporal processing ability. This aligns with previous research, which demonstrates that older people, even without peripheral hearing disorders, tend to have lower performance, and this decline often tends to increase with advancing age. 9 24

Tables 3 and 4 display multiple regression linear models assessing the predictive capacity of the age group (group), education, socioeconomic status (ABEP), temporal processing (GIN), and working memory (Corsi, TPV, Running, Digits, and Reverse Digits) in relation to listening in noise (Sentence Recognition – average and Signal/Noise ratio - S/R).

It was observed that until the fourth step, the variable group (age group) significantly predicted listening in noise, explaining 14.9% and 21.9% of the variance in Sentence Recognition - average and Sentence Recognition *-* S/R, respectively. However, with the inclusion of variables related to working memory, the age group variable lost its statistical significance. Instead, the Running Span test score emerged as a predictor of listening in noise. This finding indicates that the impact of age on listening in noise is mediated by working memory. In other words, the performance difference between groups G1 and G2 in listening in noise results not only from aging but also from the changes in working memory associated with advancing age.

Thus, the present study corroborates the findings of Gordon-Salant and Cole, 10 which showed that individuals with normal hearing and low working memory capacity, regardless of age, experience greater difficulty hearing in noise. Another study involving both young and elderly individuals with normal hearing observed that older listeners' auditory working memory capacity predicts speech recognition in challenging listening conditions. 25 However, in contrast to the latter study, the present research identified the Running Span test as a predictor of listening in noise, which assesses visual working memory. Kim et al. 25 state that auditory working memory tasks may be more sensitive in predicting speech recognition difficulties among older listeners. Conversely, the study by Zekveld et al. 26 did not support this thesis, demonstrating weak correlations between auditory working memory and speech recognition under unfavorable listening conditions.

It should be considered that our inclusion criteria for the elderly group considered normal auditory thresholds between 250 and 4k Hz, unlike the criteria for the adult group, which also included the frequencies of 6k and 8k Hz. This difference in inclusion criteria could have an impact on listening in noise, as in a study performed by Holmes and Griffiths, 27 who found that the variability of audiometric thresholds at 4 to 8 kHz explained 15% of the variance in speech performance in noise. This suggests that the audiogram contains valuable information for predicting speech understanding in real-world hearing situations, even when participants do not have clinically-impaired hearing.

Füllgrabe and Rosen 28 revealed that working memory capacity has a limited association with speech recognition in noise among younger individuals, with this association becoming more important after middle age and showing a stronger correlation with older listeners. This increased reliance on working memory capacity as age advances could be attributed to changes in the fidelity of neural encoding, resulting in poorer acoustic representation even in older individuals with normal hearing thresholds. 29 These internal changes may require more compensatory mechanisms based on working memory to activate the appropriate representations in long-term memory. Consequently, older listeners may require more cognitive strategies during speech recognition tasks in noise, while younger listeners may need more challenging conditions to be more dependent on working memory capacity. 30

Also related to these internal changes, evidence from structural neuroimaging studies has revealed that age-related atrophy in the right Heschl gyrus contributes to speech recognition difficulties in noise among older adults. 31 A more recent study published in 2021 showed that age-related structural decline in brain areas associated with hearing and cognition is linked to higher speech perception thresholds in noise in older adults. Additionally, in this same study, it was observed that the elderly with higher working memory capacity benefited more from the structural integrity of the left superior frontal gyrus, leading to improved listening recognition in noise. 8

Studies, as do the findings of the present study, suggest that speech recognition in noise for both young and older listeners may depend on working memory capacity.

The performance gap between adults and the elderly in challenging listening situations was due to age-related changes in working memory. In this study, we set out to investigate the impact of age on cognitive tasks in individuals living in developing countries and with limited education levels. However, as explained, there were limitations to this investigation.

As limitations of the present study, we can mention a small sample size, the inclusion of only female participants in the adult group, and a significant difference in education between the two groups. Further research is warranted to explore the impact of age on working memory and speech recognition in noisy environments.

## Conclusion

The present study has demonstrated that working memory is a predictor of auditory performance in noise, even among individuals with normal audiometric thresholds. This finding suggests that assessing working memory may provide valuable and complementary insights for investigation in individuals who experience difficulty hearing in unfavorable environments.
